# Micro(nano)plastics and Terrestrial Invasive Plants

**DOI:** 10.3390/toxics14030251

**Published:** 2026-03-12

**Authors:** Yanna Zhao, Jiao Sun, Fayuan Wang

**Affiliations:** College of Environment and Safety Engineering, Qingdao University of Science and Technology, Qingdao 266042, China

**Keywords:** alien plants, ecological risks, global change factors, microplastics, plant invasiveness

## Abstract

Microplastics (MPs) and nanoplastics (NPs) have emerged as pervasive contaminants across diverse environments—including soil, water, and the atmosphere—posing substantial risks to resident organisms. Concurrently, alien plant invasion represents a significant driver of environmental change, introducing considerable ecological risks to terrestrial ecosystems. Synthesizing evidence from 26 original research articles, this review examines the bidirectional interactions between micro(nano)plastics (MNPs) and terrestrial invasive plants. A growing body of evidence indicates that MNPs alter the growth and performance of both invasive and native plants. In most documented cases, MNPs appear to enhance the competitive ability of invasive plants, thereby elevating their invasion potential. However, counterexamples exist wherein MNPs strengthen the competitiveness of native plants, consequently mitigating invasion risk. These divergent outcomes are likely attributable to a suite of influencing factors, notably the characteristics of the MNPs (e.g., type, size, concentration), the specific invasive and native plant species involved, and variations in experimental conditions. Key mechanistic pathways involve MNPs-induced disturbances in soil microecology—particularly nutrient dynamics and rhizosphere microbiomes—and allelopathic interactions. Conversely, invasive plants may adsorb/absorb MNPs and subsequently modify their environmental fate and behaviors (e.g., degradation, transport). Finally, we delineate critical knowledge gaps and propose prioritized directions for future research. This review advances our understanding of the ecological risks associated with plant invasions in an era of pervasive MNP pollution and offers a scientific foundation for developing informed management strategies.

## 1. Introduction

Invasive plants refer to exotic species that can successfully colonize and proliferate in new environments, forming persistent populations that disperse widely [1]. Invasive plants exert profound and often irreversible impacts on terrestrial ecosystems worldwide. Beyond displacing native biodiversity, they fundamentally alter ecosystem processes by acting as “ecosystem engineers”—modifying soil chemistry, disturbance regimes, and nutrient cycling, which leads to degraded ecosystem functions and costly restoration challenges [2,3]. A key driver of their success is superior ecological resilience, enabling them to thrive under diverse abiotic stresses, such as drought, nutrient deficiency, and notably, soil contamination by heavy metals [4,5,6]. This tolerance often stems from integrated physiological, biochemical, and microbial strategies, such as enhanced metal detoxification and symbiotic relationships with rhizosphere microbiota (e.g., *Solidago canadensis*) [7,8].

Concurrently, microplastics (MPs) ranging from 1 μm to 5 mm and nanoplastics (NPs) smaller than 1 μm, collectively classified as micro(nano)plastics (MNPs), have emerged as pervasive environmental contaminants worldwide [9,10]. Numerous studies have confirmed the occurrence of MNPs in global soils [10] and their potential risks for terrestrial ecosystems [11,12,13]. MNPs can change soil properties, soil fertility, and the toxicity of co-occurring pollutants, causing a potential threat to soil health [13,14], and indirectly affect the performance of plants exposed to them [15,16]. Particularly, micrometer MPs and NPs can be adsorbed and absorbed via both roots and leaves, and further change plant growth, productivity, and quality [17,18]. MNPs can alter the communities of soil microbiomes and their activity, directly and indirectly, regulating their ecological functions such as nutrient cycling and greenhouse gas emissions [19,20].

Increasing evidence shows that environmental stress often facilitates plant invasion due to the superior fitness and plasticity of invaders [21,22,23]. For example, heavy metal pollution may favor plant invasion due to the higher tolerance of invasive plants compared to native plants [5]. Similarly, MNPs represent a novel and pressing anthropogenic stressor that may interact with invasion dynamics [24]. MNPs could affect the growth and invasiveness of invasive plants via direct and indirect pathways [25]. Particularly, MNPs can interact with other environmental stressors, such as toxic metals and drought [26,27], thereby amplifying or mitigating their effects on invasive plants. In turn, invasive plants may influence the environmental behaviors of MNPs through secreting allelochemicals and recruiting specific microbial communities. Thus, the interactions between MNPs and invasive plants need to be unveiled to address their combined ecological risks.

To date, several studies have focused on the effects of MPs on invasive plants [28,29,30,31,32]. However, the interactions between MNPs and invasive plants have not been systematically elucidated. As far as we know, only one mini-review briefly introduces the synergistic negative effects of MPs and invasive plants (exemplified by *S. canadensis*) on agro-ecosystems and their impacts on crop productivity [33]. To address this gap, the present review synthesizes current knowledge to critically analyze the interactions between MNPs and terrestrial invasive plants. By doing so, we aim to provide a mechanistic framework for understanding the joint ecological risks posed by these two co-occurring global change factors.

## 2. Effects of MNPs on Invasive Plants and Their Invasiveness

By searching the databases, including Google Scholar and Web of Science, a total of 26 original research papers were collected, all of which are about MPs and none involve NPs (Table 1). Among these studies, only one study examined the effects of MPs on invasive plants grown without co-existing native plants [34], which found that polylactic acid (PLA) MPs exhibited greater toxicity than polyhydroxyalkanoate (PHA) in *S. canadensis*, resulting in a range of decrease in plant height, root length, biomass, nutrient, chlorophyll content and photosynthetic capacity, but increase in antioxidative capacity. These findings support the widely acknowledged negative effects of MPs on higher plants [16,17].

The remaining 25 studies were all conducted as laboratory soil (except one with sediment) pot experiments, utilizing simulated MPs pollution to comparatively examine the responses of invasive plants and native plants to the targeted MPs. By comparing the performance of invasive plants and native plants under MPs pollution, particularly regarding plant growth status (biomass, height, etc.), it can be determined whether MPs increase or decrease the risks of plant invasions. However, the findings obtained are relatively complex, as the effects of MPs on both invasive and native plants may be either positive or negative. A successful plant invasion depends on competition between invasive and native plants, particularly the invasiveness of the invasive species and the invasion resistance of the native species. Therefore, it is necessary to compare both direction (positive/negative) and magnitude (effect size) of these effects.

### 2.1. MPs Increase the Competitive Capacity and Invasion Potential of Invasive Plants

As shown in Table 2, in most cases, MPs can enhance plant invasion potential, manifesting in the following scenarios.

(1) MPs promote invasive plants while inhibiting native plants. The earliest research in the field of MPs-plant invasion came from Rillig’s group [48], which showed that polyester (PES) fibers improved the shoot biomass of invasive *Calamagrostis* and the allelopathic *Hieracium*, but decreased the biomass of some other species. Thus, PES fibers may facilitate plant invasions. Tian et al. [52] found that biodegradable MPs reduced soil available N and P contents, while facilitated arbuscular mycorrhizal (AM) fungal colonization on the roots of the invasive *T. repens*, which prompted *T. repens* to adjust its N and P foraging strategy (e.g., increasing P absorption ratio, enhancing N and P accumulation in leaves), thereby strengthening its competitive advantage and aggravating its invasion over the native *O. corniculata*. The promoting effect of biodegradable MPs was positively correlated with their degradation degree, following an order of PHA > PLA > polybutylene succinate (PBS). Tian et al. [53] further unveiled the underlying mechanisms by which the biodegradation of MPs caused soil NO_3_^−^-N consumption, thus stimulating the colonization of AM fungi and rhizobia, which further improved the growth and competitive capacity of *T. repens*. Obviously, this mechanism may not apply to those invaders that cannot form symbiotic associations with AM fungi or rhizobia.
(2) MPs do not affect invasive plants but inhibit native plants. PE MPs decreased the biomass of native *Chenopodium album*, but did not alter the biomass of invasive *Amaranthus palmeri*, indicating that MPs pollution may facilitate plant invasion [41]. Similarly, Lozano et al. [42] demonstrated that PE particles primarily physically inhibited seed germination of native species, while having neutral effects on invasive species; and chemical additives mainly showed negative effects on natives. As invasive plants generally can have high resource use efficiency and allocation flexibility to cope with environmental changes, MPs and the plastic additives they release may promote the dominance of invasive species, posing risks to the dominance and biodiversity of native plant communities.
(3) MPs exert a stronger promoting effect on invasive plants than on native plants. Sometimes, invasive species exhibited a more sensitive growth response to MPs than native species. Deng et al. [47] found that PES fibers increased both native and invasive plants, and the promotion for invasive plants (*G. parviflora*) was more pronounced, which implies exposure to PES fibers may help *G. parviflora* to occupy unique ecological niches. Another case study is that, when added to a lake sediment, PS MPs increased the biomass of invasive submerged macrophytes more than native species [50], which may also promote plant invasion in aquatic environments. However, this study was conducted using macrophytes and sediment, which may not represent soil-terrestrial plants.
(4) MPs impose a weaker inhibitory effect on invasive plants than on native plants. Javed et al. [46] observed that a mixture of polystyrene (PS) and polypropylene (PP) MPs negatively affects the growth and performance of both plants; invasive *Sphagneticola trilobata* showed higher resistance than native *Sphagneticola calendulacea.* Okundi et al. [44] found that 1% PE MPs inhibited the growth of six invasive plants and six native plants, but invasive species showed greater biomass allocation plasticity. Low-density polyethylene (LDPE) MPs caused more significant impacts on the native species *S. decurrens* than invasive species *S. canadensis*, suggesting that MPs may promote the invasion of *S. canadensis* with a greater resistance [28]. Overall, PP and PBS MPs decreased biomass production of invasive and native plants, but increased plant invasion due to the higher biomass of invasive plants; under a fluctuating water regime, MPs even promoted the growth of invasive plants (*Paspalum dilatatum* and *Sphagneticola trilobata*) but had no effects on native plants (*P. distichum* and *S. calendulacea*) [29]. At the dose of 10%, both PLA and polyvinyl chloride (PVC) MPs had negative effects on the growth of invasive plants and native plants, but invasive species experienced less growth inhibition [31].

In addition to the scenarios mentioned above, some researchers have only examined the responses of native plants to MPs and invasive species, yet their findings still support the conclusion “MPs amplifying the risk of plant invasions”. Researchers from the same team conducted a series of studies on the effects of MPs and invasive plants on rice [35,36,37]. The results showed an overall synergistic inhibition of PE MPs and *S. canadensis* on rice growth and root development, with poorer nutrition and photosynthetic performance, higher oxidative damage, and stronger disturbed metabolites. Thereafter, they found that individual invasion legacy or MPs slightly promoted root growth, but the combination treatment strongly inhibited wheat germination and root development [38]. Similarly, a synergistic inhibition was observed between PLA MPs and invasive plant *S. canadensis* on alfalfa growth [51]. The synergistic inhibition may be ascribed to two reasons: (1) MPs may interact with the allelopathic compounds from *S. canadensis*, promoting the mobility and inhibitory effects of these compounds; and (2) MPs and *S. canadensis* invasion legacy can jointly modify soil properties and microbial communities and functions. Numerous previous studies have confirmed the alterations in soil properties and microbial communities induced by MPs [19,55,56] and the rhizosphere microbes of invasive plants exposed to MPs [31,51].

### 2.2. MPs Decrease the Competitive Capacity and Invasion Potential of Invasive Plants

Due to the diversity and heterogeneity of MPs, the characteristics of invasive and native plants, and the complexity of soil conditions, MPs do not always promote plant invasion. In some cases, MPs may mitigate plant invasions, but the outcomes are context-dependent (Table 2).

(1) MPs exert a stronger inhibitory effect on invasive plants than on native plants. Zhao et al. [45] found that PP MPs caused more biomass reductions in invasive plants than in native species, confirming a higher sensitivity of invasive plants to MPs. This finding indicates a negative effect of MPs on plant invasions. Similarly, both PE and PP MPs reduced the height and biomass of invasive *A. palmeri* and the dominant native species *Setaria viridis* and *C. album* in the community, and decreased the abundance of *A. palmeri* but increased community invasion resistance [32]. This can be ascribed to the MPs-induced changes in soil properties such as pH, P, and organic matter. Another study focused on MPs’ diversity. Fu et al. [54] discovered that the inhibition of MPs’ diversity was more pronounced for invasive plants compared to native plants, and that this may be ascribed to differences in the responses of root allocation (native plants) and thickness (invasive plants).
(2) MPs inhibit invasive plants while promoting native plants. Only one study observed such a finding. He et al. [39] found that 0.4% PE MPs decreased the growth of *Symphyotrichum subulatum* and reduced its invasion success. This is likely due to the enhancement of the selection effect: MPs increased the growth of dominant native plants (e.g., *Leonurus japonicus*) and their competitiveness over invasive species.
(3) MPs do not affect invasive plants but promote native plants. Shi et al. [40] found that PE MPs showed overall positive effects on the growth of native plants but only a marginal influence on invasive plants in fertilized soil. This suggests a potential inhibition of MPs on invasive plants. However, they still observed greater adjustment for several functional traits to PE MPs. Another study showed that PE increased the growth of native species but had no significant effects on invasive species, and enhanced the allelopathy of native *Achyranthes* to invasive species [30]. Leaf metabolome analysis showed that some up-regulated allelopathic compounds, bisdemethoxycurcumin, ethylparaben, salicin 6′-sulfate, and 5-hydroxy-3′,4′,7-trimethoxyflavone glucoside, may be involved in this enhanced allelopathy [30]. This result implies a possible mechanism that PE MPs may stimulate the allelopathic potential of native plants against invaders.
(4) MPs exert a stronger promoting effect on native plants than on invasive plants. Theoretically, when MPs promote the growth of both invasive and native plants, if the effect is weaker on the former than on the latter, they could still suppress invasion. However, this scenario has not yet been confirmed.

## 3. Mechanisms and Factors Influencing MNPs’ Effects on Invasive Plants

### 3.1. Potential Mechanisms by Which MNPs Alter Plant Invasiveness

The key mechanisms underlying the influence of MNPs on plants have been widely recognized [15,16,17], including (1) physical damage and blockage after absorption onto or absorption into roots, leaves, and seeds; (2) release of toxic plastic additives; (3) induction of the production of reactive oxygen species (ROS) and oxidative damage; (4) disturbances of soil nutrient and water availability and their uptake; (5) interacting with or acting as vectors for co-occurring pollutants (“Trojan Horse” effect); (6) alteration of soil microecology, particularly soil physico-chemical properties and rhizosphere microbiomes; and (7) interference with gene expression and metabolic profiles (e.g., root exudates).

**Figure 1:**
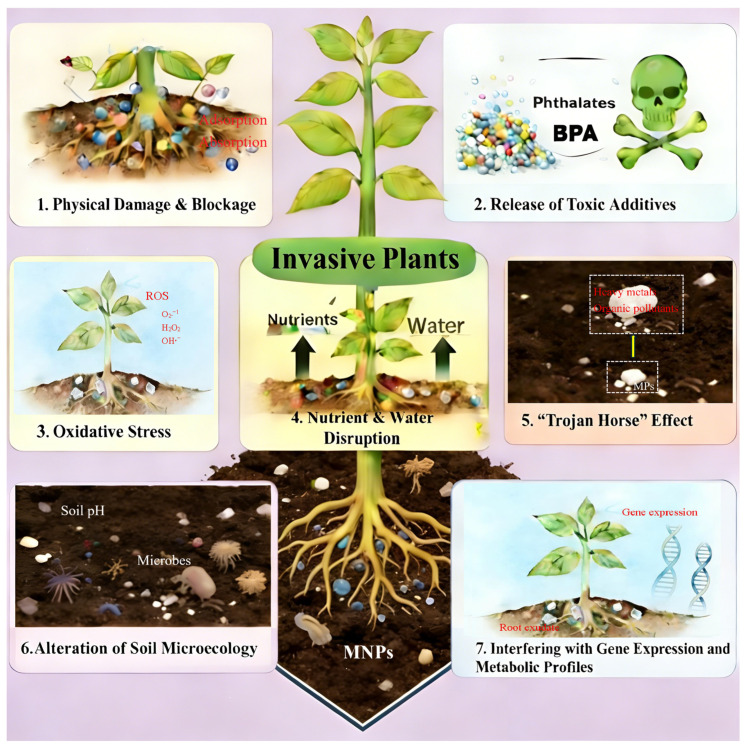
Key mechanisms by which MNPs affect invasive and native plants. Note: Microsoft 365 Copilot was used to generate the plants and soil in this figure.

Understandably, these mechanisms also apply to invasive plants co-existing with or without native plants, some of which have been confirmed (Figure 1). MPs can affect the seed germination of native and invasive plants via physical inhibition of particles and additives to some extent [42]. Another study also ascribed the different influences of plastic litter on native and invasive plants to physical (e.g., persistence) and chemical (e.g., compounds released during degradation) mechanisms [57]. Many studies have confirmed MPs-induced disturbances in soil microecology, soil nutrient and water availability, and plant mineral nutrition status [29,32,37,52,53]. According to the “Fluctuating Resource Availability Hypothesis”, invasive plants generally benefit from the presence of fluctuating available resources [58]. MNPs-induced fluctuating resource availability may be a key mechanism accounting for the impact of MPs on plant invasiveness. One typical case is that biodegradable MPs-caused soil NO_3_^−^-N pulse exacerbated the invasion of *T. repens* [53].

Oxidative damage represents one of the most common ecotoxic effects that MNPs induce in plants [17]. Anas et al. [34] found that PLA and PHA MPs both caused higher activities of antioxidative enzymes in invasive *S. canadensis* leaves and roots, indicating the occurrence of oxidative stress by MPs and an antioxidative defense capacity of this plant. ROS level in rice seedlings was not influenced by *S. canadensis* alone, but stimulated by MPs and the MP–*S. canadensis* combination [37], confirming that MPs can cause oxidative stress in native plants, thus potentially increasing invasibility. A similar finding was also observed using the *S. canadensis*–alfalfa combination [51].

The “Enhanced Mutualisms Hypothesis” posits that some invasive plants can alter the soil microbial community structure in the invaded area by enriching specific beneficial microorganisms that promote their own growth, thereby facilitating their invasion process [59]. Wang et al. [31] found that MPs caused selective enrichment of rhizosphere bacterial genera of three invasive plants, including *Arthrobacter*, *Sphingomonas*, *Microvirga*, and *Azospirillum*, which may explain why invasive plants were less inhibited than native plants. Another study showed that MPs and *S. canadensis* co-shaped rhizosphere community structure, contributing to the inhibition of alfalfa [51]. AM fungi and rhizobia that can form mutual symbionts with host plants to cope with abiotic and biotic stressors, such as nutrient deficiency, drought, and soil pathogens [60,61], have been shown to affect invasion success [62]. Recent evidence showed that MPs increased the colonization by AM fungi and rhizobia in the roots of an invasive plant (*T. repens*), helping it to overcome nutrition deficiency [52,53].

A large body of research has confirmed the interactions between MNPs and coexisting pollutants in the soil–plant system [14,17], but only a limited number of studies have focused on invasive plants versus natives [39,43,46]. Cd alone increased the growth and invasion success of *S. subulatum*, but this stimulation was reduced by MPs, indicating an antagonistic interaction with Cd on invasion success [39]. Another study showed that the effects of MPs and Cd on the invasion resistance of native species were highly dependent on plant community diversity and composition [43]. Javed et al. [46] found that MPs and nano-TiO_2_ had a synergistic interaction to favor the invasive *S. trilobata* and suppress the native *S. calendulacea*. This limited evidence suggests that MNPs can interact with co-occurring pollutants to alter their impact on plant invasions, yet the direction and magnitude remain inconclusive.

Allelopathy, as the core of the “Novel Weapons Hypothesis”, is widely used to explain the invasiveness of invasive plants over natives [63]. Invasive plants can not only directly inhibit native plants by secreting allelochemicals but also indirectly suppress the growth of native plants by altering soil physico-chemical properties and soil microbial communities. Although Iqbal et al. [37] did not identify specific allelochemicals, the leaf metabolomic analysis revealed that PE MPs reshaped the plant’s metabolic network, leading to the inference that allelopathy may be one of the underlying mechanisms of plant invasions. Conversely, PE MPs enhanced the allelopathy of native *Achyranthes* to invasive species [30]. Another study showed that polycaprolactam MPs decreased the allelopathic potential of *Iris pseudacorus* by releasing toxic caprolactam, damaging rhizosphere bacteria that down-regulate the expression of the allelopathic gene FAD2 and the synthesis of allelochemicals (e.g., palmitic acid) [64].

### 3.2. Influencing Factors

As summarized in Table 1 and Table 2, existing research presents heterogeneous outcomes regarding the effects of MPs. The variations in plant performance (for both invasive and native species), soil properties, and microbial community structure can primarily be ascribed to several key factors: the characteristics of the MPs themselves (e.g., polymer type, degradability, size, concentration, and shape), the specific plant species and community composition involved, and the experimental conditions (such as soil properties and duration). The influence of these factors has been well-documented in studies involving common plant species [15,16,17], and they appear to similarly govern the outcomes in systems involving invasive plants.

**Figure 2:**
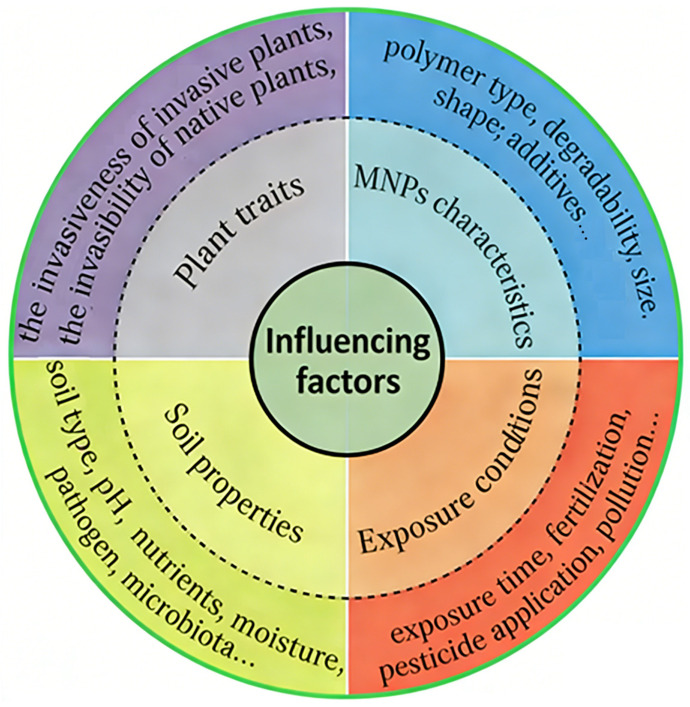
Factors modifying the effects of MNPs on invasive plants.

The polymer type of MPs determines their degradability and toxicity, which, together with their size and dosage, collectively influence their phytotoxicity (Figure 2). Zhang et al. [29] found that the inhibitory effect of PBS on total roots and fine roots was greater than that of PP, confirming different effects of biodegradable and non-degradable MPs. When applied at a dose of 10%, PLA MPs caused stronger growth inhibition in both invasive and native plants than PVC MPs [31], which may be partly ascribed to their different effects on rhizosphere microbes and activities. Although all three biodegradable MPs increased the growth and invasion over the native *O. corniculate*, the effects showed positive correlation with their degradation degree (PHA > PLA > PBS) [52,53]. Greater degradation of MPs could cause higher NO_3_^−^-N consumption and N deficiency, which may induce plants to recruit beneficial rhizosphere microbes (AM fungi and rhizobia) to cope with nutrient deficiency [53]. Meng et al. [32] observed negative effects of PE and PP MPs on invasive *A. palmeri*, but the effect size varied with the polymer type and dose, with more pronounced effects from PP and low dose. Li et al. [28] also found that the effects of LDPE MPs on native and invasive plants were dependent on MP shape, dose, and their interactions. Fu et al. [54] showed that a higher diversity of MPs caused stronger growth inhibition, because of the inclusion of MPs with higher toxic effects, such as PBS, polycaprolactone, and PLA, and their pairwise interactions. In real soils, MPs generally exhibit diversity in terms of type, shape, concentration, size, and plastic additives, which partly explain the various results observed in different studies.

The success of plant invasions depends on both the invasiveness of the invasive plants and the invasibility of the native plants. Shi et al. [40] observed variable growth responses in different invasive plants to PE MPs: positive in *Phytolacca americana*, but negative in *Bidens pilosa* and *Chromolaena odorata*. He et al. [39] found that PE MPs alone reduced the invasion success of *S. subulatum*, and the effect increased with increasing native community diversity, which indicates that a high biodiversity should have a higher resistance to invasion. Similarly, Javed et al. [43] found that invasion resistance increased with native diversity, which was further mediated by MPs.

Biotic factors such as pathogens and herbivores influence the response of plants to MPs. Anas et al. [34] found that the combined stress of MPs (PLA or PHA) and a fungal pathogen (*Rhizoctonia solani*) caused stronger inhibition in *S. canadensis* growth. Meanwhile, this invasive plant exhibited an adaptive capacity by activating antioxidative and extracellular enzymatic mechanisms. Furthermore, this fungal pathogen intensified the inhibition of *S. canadensis* and PLA MPs on alfalfa seedling growth [51]. Only one study explored the interaction of herbivores and MPs. Okundi et al. [44] observed an antagonistic interaction between PE MPs and herbivory by *Helicoverpa armigera* on both invasive and native plants. The interactions between different stressors on plants may be complex, which are not always synergistic or additive, because plants may cope with multifactorial stress conditions through common molecular and biochemical mechanisms [65]. In addition, both PE MPs and invasive plants significantly changed the metabolomic profiling in rice leaves, such as down-regulated saccharides and up-regulated citric acid [37], which may further influence the feeding preferences of herbivores.

Soil water is crucial for the growth of all plants. As MPs can alter water retention and availability in soil, their effects vary with drought status and water regimes. Lozano et al. [48] showed that PES fibers reduced soil bulk density and increased soil water-holding capacity, thereby counteracting the adverse effects of drought on the growth of invasive and native plants. Similarly, Deng et al. [47] found that drought negatively affected the growth of invasive *G. parviflora*, while microfibers showed a promoting effect, indicating an antagonistic interaction between them. A possible explanation can be ascribed to the positive influence of PES fibers on soil aeration, bulk density, and water retention [48,66]. Differently, Zhang et al. [29] found that PBS MPs produced negative effects on invasive plants under a constant water regime but positive effects under a fluctuating water regime.

Other environmental change factors may interact with MPs to produce synergistic or antagonistic outcomes. Cd alone increased the growth and invasion success of *S. subulatum*, but this stimulation was reduced by MPs [39], indicating an antagonistic interaction with Cd on invasion. Wang et al. [41] found that the detrimental effects of MPs were mitigated by N deposition, implying an antagonistic interaction between MPs and N deposition. This can be ascribed to the enhanced niche differentiation of plant communities and differences in resource utilization among different species. In addition, N may reduce biotic filtering caused by high niche overlap and directly provide nutrients for plants, mitigating MPs-induced N deficiency. Although all the tested environmental change factors (drought, salinity, eutrophication, heat wave, MPs, and herbicides) had negative effects on invasive plants, drought and salinity generally dominated the interactions (synergistic or antagonistic) among various combinations [45]. All these factors, including MPs and species invasion, are considered global change drivers [67], and their combined effects warrant further investigation.

Agricultural practices such as fertilization and pesticide application affect both invasive and native plants/crops. Only two studies have focused on this topic. Shi et al. [40] found that PE MPs promoted native plant growth after fertilization, but not for invasive species, thus inhibiting the invasion success in fertilized agricultural fields. Javed et al. [46] revealed that MPs and nanopesticide (TiO_2_) exhibited stronger inhibitory effects on the growth of native *S. calendulacea*, as well as greater interference with soil nutrients, enzyme activities, and greenhouse gas emissions, compared to their effects on invasive *S. trilobata*. Moreover, the combined treatment of MPs and nanopesticide showed more significant effects than single treatment with MPs or nanopesticide alone. It is noteworthy that agricultural practices such as fertilization and pesticides can interact with MPs in agricultural soils [68,69]. The performance of invasive plants in crop fields with abundant MNPs may need more attention.

## 4. Potential Effects of Invasive Plants on MNPs

### 4.1. Accumulation and Translocation of MNPs

Previous evidence has shown that MNPs ranging from nanoscale to a few micrometers can enter plant roots and/or further transfer to aboveground tissues, and also enter leaves through stomatal pathways and subsequently undergo downward translocation [17,18,70]. Although the evidence is lacking to date, terrestrial invasive plants may accumulate MNPs (particularly NPs and sub-micrometer MPs) via roots and/or leaves due to their pollution adaptability and efficient resource utilization capabilities. Since both invasive and native plants can accumulate MNPs, when they grow together in MNPs-polluted sites, they may influence each other’s uptake of MPs, potentially impacting the quality and safety of agricultural products. In addition, the bioaccumulation of MNPs by invasive plants may provide a phytoremediation strategy for MNPs-polluted soil [71]. After harvest, measures such as incineration and pyrolysis may be taken for the safe treatment of the plant tissues containing MNPs. Therefore, the uptake and translocation of MNPs by invasive plants, as well as their impact on the bioaccumulation of MNPs by native plants, warrant in-depth investigation.

### 4.2. Environmental Fate and Behaviors of Soil MNPs

The generation, migration, aging, and degradation of MNPs in soil are influenced by a combination of physical, chemical, and biological processes [72,73,74]. For example, soil biota (fauna and microorganisms) and soil constituents (e.g., acids and alkalis) can contribute to the fragmentation and degradation of MNPs [72,74]. A recent study showed that soybean rhizosphere markedly accelerates the degradation of large PBAT MPs, which can be ascribed to the altered soil physico-chemical properties (e.g., increased soil aggregation and soil pH) and higher microbial biomass and activity [75]. Planting radish mitigated the disturbance of MPs (PS, PE, and PP) on soil ecological functions via rhizosphere effects [76]. Although no direct evidence has been confirmed, invasive plants may—either independently or in conjunction with native plants—alter soil structure and modify the soil microenvironment through the secretion of root exudates (e.g., allelopathic compounds) and the recruitment of specialized soil biota communities [77,78]. These changes may further trigger a cascade of effects on the generation, aging, fragmentation, migration, and degradation of MNPs. Rhizoremediation using invasive plants and their associated rhizosphere microbes may offer a viable strategy for addressing soil MNP pollution. Similarly, plant roots can alter the migration of MNPs in soil through direct physical entanglement, as well as indirectly by modifying soil structure and water conditions. Li et al. [79] found that crop roots tended to move MPs upwards or retain them in soil layers, but these effects varied with crop species. Given that invasive plants exhibit diverse root architectures—ranging from deep taproots to dense fibrous root systems—it is reasonable to hypothesize that their establishment may similarly alter the migration of MNPs in invaded soils.

It has been widely acknowledged that invasive plants can reduce biodiversity and disrupt soil structure [80], increasing soil erosion [81], which may enhance the likelihood of MNPs in surface soil being transported into the atmosphere by wind [82] or into aquatic environments by surface runoff [83]. In addition, in invaded ecosystems, the reduced presence of natural enemies of invasive plants may alter the risk of MNPs’ transfer through food chains.

## 5. Conclusions and Future Perspectives

This review shows that MNPs can modify the invasiveness of invasive plants. In most documented cases, MNPs increase the competitive advantage of invasive plants, thereby elevating their invasion potential. However, counterexamples exist wherein MNPs fortify the competitiveness of native species, leading to a mitigated invasion risk. These context-dependent outcomes are primarily attributed to: (1) the traits of MNPs (e.g., type, size, shape, dose, and degradability); (2) the biological traits of the plants (e.g., the invasiveness of alien species and the invasibility resistance of native plants); and (3) environmental mediators, including biotic factors (e.g., soil pathogens and herbivores) and abiotic conditions (e.g., soil moisture, nutrient status, and agricultural practices). The underlying mechanisms involve both direct and indirect pathways. MNPs can inflict physical damage, induce oxidative stress, and leach toxic additives. Indirectly, they alter soil microecology—modifying physico-chemical properties and restructuring rhizosphere microbial communities—which in turn interferes with nutrient dynamics, root exudation, and allelopathic interactions. Furthermore, MNPs can act as vectors for co-occurring pollutants, compounding their ecological impact. Conversely, invasive plants are not passive recipients; they potentially adsorb/absorb MNPs and influence MNPs’ fate through effects on degradation, fragmentation, and soil transport processes.

However, there are still large knowledge gaps in the research of MNPs-invasive plants interactions that should be addressed in future work. We recommend several future research priorities below.

(1) Mechanistic research on MNPs’ effects on plant invasions: Future studies should employ multi-omics technologies and integrate plant invasion hypotheses to elucidate the mechanisms through which MNPs promote or inhibit plant invasions.
(2) Ecological risks posed by MNPs in plant invasions: The potential of MNPs to directly or indirectly amplify the adverse effects of plant invasions on soil processes, microbial diversity, plant diversity, ecosystem stability, and functions is critically underexplored.
(3) A broader range of subjects: Future research must consider the diversity of MNPs types found in soils and their environmentally realistic concentrations. A significant research focus should be placed on NPs and tire wear particles. While plant invasion is a global change driver, current research involves only a limited number of invasive species. For instance, according to the Global Register of Introduced and Invasive Species Dataset (https://griis.org/), the United States has 4337 registered invasive plant species, while China has 594. As the high species diversity of native plants can enhance resistance to invasion, future studies should incorporate a greater variety of native species and different native–invasive plant combinations.
(4) Multiple-scale studies: So far, only one field study has investigated the abundance of MPs in a wetland with invasive *Spartina alterniflora* [24]. Current research predominantly relies on indoor pot experiments, which inadequately simulate field conditions. Given the variable degradability of different MNPs, their long-term ecological impacts require greater attention.
(5) Combined effects of MNPs and other environmental stressors: MNPs may interact with other environmental stressors to produce synergistic or antagonistic consequences for plant invasions and the invaded sites, which needs to be unveiled.
(6) Impact of invasive plants on MNPs: Future work should strengthen investigation into how invasive plants influence the environmental fate and behaviors of MNPs, including their formation, aging, degradation, and migration, with particular emphasis on the mechanisms of uptake, translocation, and associated food chain risks.
(7) Interactions between airborne MNPs and invasive plants: Current research focuses solely on soil MNPs. However, MPs are ubiquitous in the atmosphere. They can not only enter into the soil and affect root systems but also deposit directly on leaf surfaces, influencing leaf functions (e.g., photosynthesis and transpiration). Moreover, MNPs of certain particle sizes can enter leaves, accumulating within plants. All these processes can affect the performance of invasive plants and the environmental fate of airborne MNPs.

## Figures and Tables

**Table 1:** Effects of MPs on invasive and native plants.

MP Characteristics	MP Dose (*w*/*w*)	Substrate	Invasive Plant	Native Plant	Duration	Key Findings	Reference
PE mixture (pellets:fragments:fibers = 3:4:3); 0.6–1.0 mm (pellets & fragments); length: <2.0 mm, diameter: 0.2 mm (fibers); unknown aging status	0.5%	Soil from Jiangsu Univ. campus	*S. canadensis*	*Oryza sativa*	50 & 80 days after transplanting	Combined invasion and MPs were most harmful; reduced biomass, photosynthesis, and altered antioxidant enzymes; invasion alone had least impact.	[35]
			*S. canadensis*	*Oryza sativa*	50 & 80 days	Combined invasion and MPs had the most adverse effects on rice root traits; MPs alone increased root diameter; invasion alone had the least impact.	[36]
			*S. canadensis*	*Oryza sativa*	80 days	Combined invasion and MPs were the most harmful; reduced leaf biomass, C/N/P content, altered antioxidant enzymes, and metabolomic profile; synergistic negative impact on rice.	[37]
PE mixture (pellets:fragments:fibers = 3:4:3); 0.5–1.0 mm; unknown aging status	0.4%	Soil from Jiangsu Univ. greenspace	*S. canadensis* (invasion legacy effect)	*Triticum aestivum*	30 days	Individual legacy or MPs slightly promoted root growth; combined treatment strongly inhibited germination and root development.	[38]
HDPE powder; 150 μm; unknown size range and aging status	0.4%	Soil:peat:sand (3:1:1) mixture; loamy sand, pH 6.2	*Symphyotrichum subulatum*	Four native species combinations	12 weeks	MPs alone reduced invasion success; Cd alone increased invasive plant biomass; MPs inhibited Cd’s promotion of invasion.	[39]
LDPE; 0.45–1.00 mm (pellets & fragments); unknown aging status	0.5%, 2.0%	Loamy sand, pH 7.69	*S. canadensis*	*Solidago decurrens*	6 months	MPs affected soil properties and native plant growth (biomass, antioxidant enzymes) more than invasive plants. Invasive *S. canadensis* showed greater resistance to MPs, suggesting MPs may promote its invasion by differentially impacting native competitors.	[28]
PE; 150 μm; unknown shape, size range, and aging status	2% of the soil fresh weight	Soil:sand (2:1) mixture	5 alien species	5 native species	~4 months	Under nutrient addition, microplastics promoted biomass production of native plants but had marginal influence on alien plants. Alien plants exhibited quicker adjustment in functional traits (e.g., SLA and RMF) in response to nutrients and MPs.	[40]
PE powder; 13 μm (diameter); unknown size range and aging status	0.7%	Sandy loam, pH 4.95	*Amaranthus spinosus*	*Achyranthes bidentata*	3 months	PE increased biomass of both species under both competitive and non-competitive conditions; enhanced allelopathy of native *Achyranthes*; PE altered leaf metabolome; enhanced allelopathic potential of native plant against invaders.	[30]
LDPE pellets; ~150 μm (diameter); unknown aging status	0.1%, 0.5%, 1%	Coastal wetland soil	*A. palmeri*	*C. album*, etc.	90 days	MPs reduced morphological traits of invaded plant communities; invasive species showed higher pollution tolerance than natives; nitrogen addition mitigated negative effects of MPs on diversity and stability.	[41]
LDPE films; 0.07 mm (thickness), size ≈ 1 mm^2^ (shredded); UV-aged	0.4%	Sandy loam from grassland, pH 6.66	*Bunias orientalis*, *Tanacetum vulgare*, *C. epigejos*	*Achillea millefolium*, *Dactylis glomerata*, *Daucus carota*, *T. repens*	3–5 weeks	Plastic particles primarily physically inhibited seed germination of native species (reduced germination, velocity, and synchrony), while having neutral effects on invasive species; chemical additives had secondary, mainly negative effects on native species.	[42]
PE particles; 150 μm; unknown size range and aging status	0.4%	Soil:peat:sand (3:1:1) mixture; loamy sand, pH 6.2	*S. subulatum*, *Sphagneticola trilobata*	*Lactuca indica*, *Plantago asiatica*, etc.	16 weeks	MPs and Cd significantly affected biomass of both native and invasive species; invasion resistance increased with native diversity; *S. trilobata* showed stronger growth and soil impact.	[43]
PE powder; 100 mesh; unknown aging status	1%	Sand:soil (2:1) mixture; mountain soil, pH 4.53	6 invasive species (e.g., *S. canadensis*, *Crotalaria pallida*)	6 native species (e.g., *Achyranthes* *bidentata*, *Lolium perenne*)	12 weeks	Native plants were more affected by PE MPs than invasive plants; antagonistic interaction with herbivory; invasive plants showed greater biomass allocation plasticity.	[44]
LDPE and PP pellets; ~150 μm (diameter); unknown aging status	0.1%, 0.5%, 1%	Coastal saline soil	*Amaranthus palmeri*	*Setaria viridis*, *Chenopodium album*, etc.	Not explicitly stated	Overall, MPs reduced biomass and increased invasion resistance but reduced community stability; PP had greater negative impacts than PE.	[32]
PP fibers; unknown size and aging status	0.2%	Sand:vermiculite:yellow–brown soil (3:3:2) mixture	10 invasive-native species pairs	10 invasive-native species pairs	86 days	Invasive species were more sensitive to increasing environmental factors than native ones; drought and salinity were key drivers of biomass reduction; multiple stressors led to greater negative effects.	[45]
PP, PBS; beads: 4–5 mm; powder: 125–150 μm; unknown aging status	5% (*v*/*v*)	Vermiculite: peat = 1:1	4 invasive species (e.g., *Paspalum dilatatum*)	4 congeneric native species	60 days	Under fluctuating water regime, MPs (especially degradable PBS) promoted biomass and fine root growth in invasive species, enhanced invasiveness; reduced photosynthetic efficiency in native species.	[29]
PS:PP (1:1) mixture; unknown shape, size and aging status	3.96 g/kg soil	Clay-loam, pH 6.00–6.25	*S. trilobata*	*Sphagneticola calendulacea*	12 weeks	Invasive species were more resistant to MPs + nanopesticides; soil microbial activity reduced more in native eco-community.	[46]
PES fibers; 3.0 mm (length), 0.030 mm (diameter); unknown aging status	0.4%	Field soil from Shenyang	*Galinsoga parviflora*	*Bidens bipinnata*, *Plantago depressa*, *Medicago sativa*, *Glechoma longituba*	2 months	Microfibers increased shoot and root biomass, especially for invasive *G. parviflora*; Drought reduced biomass; UV-B effects varied by species; microfibers alleviated drought stress.	[47]
PES fibers; 1.28 ± 0.03 mm (length), ~30 μm (diameter); unknown aging status	0.4%	Sandy loam, pH 6.66	*Calamagrostis epigejos*	6 species	2 months	Microfibers increased root and shoot biomass at community level; increased dominance of invasive *Calamagrostis* and allelopathic *Hieracium*; drought decreased biomass; microfibers reduced soil bulk density, improved aeration; altered community structure and evenness.	[48]
PVC powder; <250 μm; unknown aging status	1%	Soil:sand = 4:6	*Senecio inaequidens*	*Centaurea cyanus*	60 days	PVC MPs negatively affected plant growth (height and width) in both species. MPs reduced photosynthetic efficiency (Fv/Fm) in *C. cyanus* and delayed leaf phenology in *S. inaequidens*.	[49]
PS; 48 μm; unknown shape, size range, and aging status	0.5%, 2.5%	Sediment from Donghu Lake	*Cabomba caroliniana*, *Egeria densa*, *Elodea nuttallii*	*Hydrilla verticillata*, *Vallisneria natans*, *Potamogeton wrightii*, *Myriophyllum spicatum*	13 weeks	Invasive species biomass increased more than native under MPs; DNF rates increased with MPs; MPs altered sediment microbial community; DNF gained competitive advantage over DNRA; decoupling of DNF and ANA observed.	[50]
PLA particles; 150–185 μm; unknown aging status	1%	Farmland soil	*S. canadensis*	*Medicago sativa*	4 months	MPs, invasive species, and fungal pathogens significantly reduced alfalfa growth, altered rhizospheric enzyme activities and microbial community structure, threatening grassland ecosystem functionality.	[51]
PHA, PLA, PBS; powders; 0.15 mm; unknown size range and aging status	5%	Black soil:sand (3:1, *v*/*v*) mixtrue	*Trifolium repens*	*Oxalis corniculata*	30 days	Biodegradable MPs increased the growth of invasive *T. repens*, which had higher AM fungal colonization, enhancing N and P acquisition; thereby strengthening its competitive advantage and aggravating its invasion over the native *O. corniculata*.	[52]
PHA, PLA, PBS; <0.15 mm; unknown shape and aging status	1%, 5%	Soil:sand (3:1) mixture	*T. repens*	*Oxalis corniculata*	60 days	Biodegradable MPs consumption of soil NO_3_^−^-N promotes AM fungal and rhizobia colonization, triggering NO_3_^−^-N pulse supply and enhancing competitiveness and invasion of *T. repens*.	[53]
PLA, PHA; particles; 150–185 μm; unknown aging status	1%	Forest soil from Zhenjiang, Jiangsu, pH 6.55	*S. canadensis*	None	Not explicitly stated	PLA exhibited greater toxicity than PHAs, reducing plant height, root length, and biomass, and impairing the maximum quantum yield of PSII. Under combined stress of MPs and pathogens, plants activated adaptive antioxidative and extracellular enzymatic mechanisms.	[34]
PLA, PVC; particles; 155–180 μm; unknown aging status	10%	Farm field soil, pH 8.70	*Galinsoga quadriradiata*, *Erigeron canadensis*, *Erigeron annuus*	*Artemisia dubia*, *Artemisia lavandulifolia*, *Artemisia vestita*	60 days	Invasive plants were less inhibited by MPs; enriched keystone taxa (*Arthrobacter*, *Sphingomonas*) enhanced stress tolerance.	[31]
Single or combinations of 12 types of MPs: PBS, polycaprolactone (PCL), PHA, PHB, PLA, polybutylene adipate-co-Terephthalate (PBAT), ethylene-vinyl acetate (EVA), polyamide 66 (PA66), PET, polyoxymethylene (POM), PVC, PP; powder; 150 or 180 μm	3% (*v*/*v*)	Sand:vermiculite (1:1) mixture	8 invasive species	8 native species	10 weeks	Growth suppression increased with more microplastic types; invasive species were more suppressed than native ones; invasive species showed greater increase in root thickness.	[54]

**Table 2:** The impact of MPs on invasive and native plants and invasion risks.

**Invasive Plants**	**Native Plants**	**Invasion Risk**	**Reference**
↑	↓	+	[48,52,53]
ns	↓	+	[41,42]
↑↑	↑	+	[47,50]
↓	↓↓	+	[28,29,31,44,46]
↓↓	↓	−	[32,45,49,54]
↓	↑	−	[39]
ns	↑	−	[30,40]
↑	↑↑	−	?

Note: “↑” and “↓” represent promotion and inhibition of MPs on plants, respectively; “↑↑” and “↓↓” represent stronger promotion and inhibition of MPs on plants, respectively; + and − represent increase and decrease in invasion risks, respectively. “ns” means non-significant effects; “?” means unconfirmed or no literature found.

## Data Availability

No new data were created or analyzed in this study. Data sharing is not applicable to this article.
